# *In vivo* efficacy of macrocyclic lactones on goat farms – pour-on *vs* injectable application

**DOI:** 10.2478/jvetres-2025-0025

**Published:** 2025-04-19

**Authors:** Michal Babják, Alžbeta Königová, Tetiana A. Kuzmina, Marián Várady

**Affiliations:** 1 Laboratory of Therapy of Parasitic Infections, Institute of Parasitology of the Slovak Academy of Science, 040 01 Košice, Slovak Republic; 2 I. I. Schmalhausen Institute of Zoology of the National Academy of Sciences of Ukraine, Kyiv, 01054, Ukraine

**Keywords:** goats, drug resistance, *Haemonchus contortus*, eprinomectin, ivermectin

## Abstract

**Introduction:**

Pour-on eprinomectin (EPN) is the only anthelmintic currently available that is approved for use in lactating goats with a zero-day milk withdrawal period. The aim of this study was to compare the efficacy of the pour-on form of EPN with the efficacy of the injectable version of ivermectin (IVM) on goat farms in Slovakia.

**Material and Methods:**

The study was conducted from September to December 2023 on eight dairy-goat farms. The goats on six farms were divided into two groups of 10 animals each. The first group was administered pour-on EPN at the recommended dose for sheep and goats, and the second group was treated with injectable IVM at a dose 1.5-fold higher than the recommended dose for sheep. The goats on the remaining two farms were treated only with EPN. Efficacy was determined using the *in vivo* faecal egg count reduction test and the *in vitro* larval development test (LDT). Infectious third-stage (L3) larvae were harvested before and 14 days after treatment and were assigned to species/genus based on morphology.

**Results:**

The percent reductions for IVM and EPN ranged from 80.8 to 93.6 and 51.0 to 96.6, respectively. A substantially higher egg reduction on the 14^th^ day post treatment of 20 to 30% was noted in the groups on three farms treated with injectable IVM. The reduction differed little between the drugs on the other three farms (1–3%). Infectious L3 larvae in the LDT were observed at concentrations equal to or higher than the minimum inhibitory concentration of 21.6 ng/mL on all farms. *Haemonchus contortus* dominated on the 14^th^ day post treatment with both drugs on all farms.

**Conclusion:**

This study is the first to report reduced efficacy of the pour-on form of EPN in goat herds in Slovakia.

## Introduction

Small ruminants are primarily kept for milk and meat production. Residues remaining in these products after anthelmintic treatment are therefore one of the most limiting factors in this industry. Establishing an appropriate treatment scheme is becoming more complicated as cases of anthelmintic resistance increase. Farmers and veterinarians in central Europe still only have a choice of the benzimidazole (BZ) or macrocyclic lactone (ML) classes of anthelmintics. Levamisole preparations are difficult to purchase on the local market and may be of limited use in small ruminants because of the narrow margin of safety. The application of levamisole in the last month of pregnancy is not recommended. The administration of BZs is also of limited efficacy because cases of infection with BZ-resistant strains of gastrointestinal nematodes (GINs) have become common in this region because of the frequent use of these drugs (1, 21, 22, 30), so their efficacy is very low on many farms of small ruminants.

The injectable form of ivermectin (IVM) is the most accessible treatment from the ML class, the application of which is restricted to sheep and goats producing milk for human consumption. The lipophilic features of IVM predetermine its deposition to the fatty tissues of the body. Repeated treatment of sheep and goats with IVM leads to an accumulation of residues in the milk fat, which forces farmers to condemn milk, causing them financial losses. The inclusion of IVM in the treatment scheme for dairy breeds which lactate for most of the year is therefore difficult. The 2016 launch of the ML-class drug eprinomectin (EPN) on the market as the only active substance without withdrawal periods for milk and authorised by the European Union for administration in goats initially seemed to be a solution to this issue. A marketing authorisation was established for topical application, and expectations for its use in dairy sheep and goats were high. However, the insufficient *in vivo* efficacy of topical (“pour-on”) applications of EPN on goat farms at the recommended dose of 1 mg/kg was detected in a survey in Switzerland (24) where EPN had been registered for goats since 2004. The dose of EPN for administration to sheep and goats had already been enlarged from one of 0.5 mg/kg intended primarily for pour-on use in cattle (8, 14). The results of this first comprehensive *in vivo* study in Switzerland served as an early warning. Case reports of reduced efficacy of the pour-on formulation of EPN in sheep and goats were subsequently published in France (2, 3, 17), Poland (23) and Austria (15).

Eprinomectin was used off-label in many countries before it gained marketing authorisation for small ruminants, and the pour-on formulation of EPN was commonly administered orally (7). Pour-on EPN intended for small ruminants is difficult to obtain and its retail price is financially prohibitive in Slovakia; hence, we assumed that this formulation had never been applied on many goat farms. The main aim of this study was to compare, for the first time, the efficacy of pour-on EPN with the efficacy of the most frequently used injectable IVM formulation on goat farms in Slovakia. The secondary aims were to compare the results of the *in vivo* faecal egg count reduction test (FECRT) with the data obtained from the *in vitro* larval development test (LDT) and to examine the morphology of third-stage GIN larvae (L3) before and after treatment on each farm.

## Material and Methods

### Study design

The study was conducted in September– December 2023 on 160 goats from eight dairy goat farms. The farms were situated in eastern (n = 3), central (n = 3) and western (n = 2) Slovakia. The herds varied in size between 30 and 296 animals and consisted mainly of White Shorthaired, Brown Shorthaired and Alpine goats. The age of the treated animals ranged from 1 to 8 years. The goats on six of the farms were divided into two groups of 10 animals each based on herd size and farmer consent. The first group was administered with EPN (EPRINEX Multi; Boehringer Ingelheim, Ingelheim, Germany) at the recommended dose for sheep and goats (1 mg/kg body weight), and the second group was treated with injectable IVM (Ivomec; Merial, now Boehringer Ingelheim) at a dose 1.5-fold higher (0.3 mg/kg body weight) than the dose recommended for sheep (0.2 mg/kg body weight). The goats on the remaining two farms were treated only with EPN (1 mg/kg body weight). Before administration of the drug, each animal was weighed on a scale, and the dose was individually calculated based on its weight. The goats had not been treated with any anthelmintics for at least two months prior to the study. Faecal samples were collected from each animal directly from the rectum on the day of treatment and on the 14^th^ day after treatment. The number of GIN eggs per gram of faeces (EPG) was determined 24 hours after sampling using a modified McMaster technique with a sensitivity of 50 EPG (4).

### Faecal egg count reduction test (FECRT)

The FECRT was carried out following the guidance of the World Association for the Advancement of Veterinary Parasitology (WAAVP) 4, 5). Individual efficacies of EPN and IVM before and after treatment were evaluated using a Bayesian hierarchical model and the eggCounts 2.3.2 package in R version 4.3.3 (11, 26), as described by Wang *et al*. (29). The results were interpreted following the WAAVP guidelines (Table 1) (6).

**Table 1.** Interpretation of World Association for the Advancement of Veterinary Parasitology guidelines for faecal egg count reduction test results

Efficacy	Results
Reduced	FECR% < 95% confidence interval and <90%
Doubtful	Either FECR% < 95% or lower limit of the 95% confidence interval or <90%
Normal	FECR% ≥ 95% and lower limit of the 95% confidence interval and ≥ 90%

1 FECR% – faecal egg count reduction

### Larval development test (LDT)

The LDT was performed in two replicates for each farm as described by Hubert and Kerboeuf (16) with the modifications described by Várady *et al*. (32). The GIN eggs obtained from the goat faeces were incubated for 7 days at 28°C in 96-well microtitre plates at 12 concentrations of IVM aglycone (0.084–173.6 ng/mL) (Ivermectin aglycone; Tebubio, Le Perray-en-Yvelines, France) with culture medium (yeast extract with Earle’s balanced salt solution and a physiological salt solution). The incubation was terminated by adding 10 μL of an iodine solution, and the proportions of eggs and first-stage, second-stage and third-stage larvae were determined for each concentration. The minimum inhibitory concentration (MIC) of 21.6 ng/mL (9) was used to evaluate the results. The presence of resistant GINs was suspected on a farm if L3 were found at an IVM concentration equal to or higher than the MIC.

### Morphological identification of L3

Larvae in the L3 stage were harvested from incubated faecal samples collected before treatment and on the 14^th^ day after treatment using the Baermann technique. One hundred larvae from each treated group of goats from all farms were assigned to species/genus level based on their morphological characteristics as described by Van Wyk and Mayhew (31).

## Results

In accordance with the WAAVP guidelines (Table 1), the efficacy of the injectable form of IVM was reduced on all farms (6/6), and that of the pour-on EPN on 87.5% of the farms (7/8). The percentage of EPG reduction ranged from 80.8 to 93.6 for IVM and from 51.0 to 96.6 for EPN. Three farms had a substantially higher reduction in EPG on the 14^th^ day of 20% to 30% in the groups treated with injectable IVM. The EPG reduction differed little between the two anthelmintics on the other three farms and was 1–3%.

On the day of treatment, the EPG counts across all farms and groups ranged from 150 to 8,150, and the average was above 400 in all treated groups of goats, except for those in one group on one farm. The total EPG values and results of the FECRT are summarised in Table 2. The results of the FECRT and LDT were in agreement. Infectious L3 were observed at IVM concentrations equal to or higher than the MIC on all farms (Table 3). All infectious L3 larvae detected in the LDT at IVM concentrations equal to or higher than the MIC were identified as *Haemonchus contortus*. The mean number of L3 was highest in LDTs for farms where the *in vivo* efficacy of both drugs was below 90%. Before treatment, *Teladorsagia circumcincta* was the predominant species on 75% of the farms (6/8) and *Haemonchus contortus* was the most prevalent species on the other 25% (2/8). *Trichostrongylus* spp. and *Oesophagostomum*/*Chabertia* spp. were also detected in lower proportions. *Haemonchus contortus* was the only species of GIN detected in the IVM-treated groups on all farms on the 14^th^ day. This nematode was also the dominant species on this day in the EPN groups, but *T. circumcincta* was identified at 3% and 5% prevalence on the two farms with the lowest *in vivo* efficacy of EPN. The proportions of the GINs by species before and after treatment for each farm are presented in Table 4.

**Table 2.** Results of the *in vivo* faecal egg count reduction test for pour-on eprinomectin and injectable ivermectin on eight goat farms

Farm	Eggs per gram	Pour-on eprinomectin				Injectable ivermectin	
		D0	D14	FECR% (95% CI lower and upper bounds)	D0	D14	FECR% (95% CI lower and upper bounds)
1	Mean ± SD	725 ± 861.01	20.00 ± 33.16	96.60 (90, 99)	470.00 ± 2 61.91	30.00 ± 45.82	93.60 (84, 98)
	Min-max	150–3,100	0–100		150–1,000	0–150	
2	Mean ± SD	262.50 ± 143.06	125.00 ± 75.00	59.20 (20, 86)	710.00 ± 339.70	130.00 ± 143.52	80.80 (47, 95)
	Min-max	150–500	0–200		150–1,100	0–350	
3	Mean ± SD	2,810.00 ± 1,370.36	485.00 ± 597.93	87.10 (77, 93)	2,261.11 ± 2,142.65	288.88 ± 334.81	89.20 (80, 95)
	Min-max	1400–5,300	0–2,000		800–8,150	0–1000	
4	Mean ± SD	1,495.00 ± 720.57	100.00 ± 94.86	93.50 (87, 97)	1,325.00 ± 835.16	75.00 ± 93.54	94.70 (87, 98)
	Min-max	250–2,550	0–300		350–2,950	0–250	
5	Mean ± SD	970.00 ± 772.39	430.00 ± 448.99	63.50 (36, 81)	545.00 ± 396.51	40.00 ± 76.81	92.30 (85, 96)
	Min-max	150–2,450	0–1,300		150–1,400	0–250	
6	Mean ± SD	1,170.00 ± 506.55	430.00 ± 289.13	65.40 (39, 84)	1,155.55 ± 981.33	116.66 ± 97.18	88.00 (73, 94)
	Min-max	250–2,150	0–750		200–3,550	0–350	
7	Mean ± SD	1,711.11 ± 1,023.27	694.44 ± 381.84	51.00 (27, 69)	N/A	N/A	N/A
	Min-max	350–3,350	0–1,300				
8	Mean ± SD	612.50 ± 544.14	150.00 ± 141.42	69.40 (37, 91)	N/A	N/A	N/A
	Min-max	200–1,950	0–400				

1 D0 – the day of treatment; D14 – the 14^th^ day after treatment; FECR% – percentage of faecal egg count reduction; CI – confidence interval; SD – standard deviation; N/A – not assessed

**Table 3.** The mean numbers of infective third-stage larvae at ivermectin concentrations equal to or higher than the minimum inhibitory concentration of 21.6 ng/mL for the eight goat farms

Farm	Mean number of L3 larvae ± SD at concentrations ≥21.6 ng/mL
1	3.50 ± 4.79
2	24.12 ± 4.42
3	22.75 ± 7.17
4	4.00 ± 1.87
5	5.75 ± 1.47
6	23.00 ± 11.92
7	5.25 ± 1.08
8	8.12 ± 2.84

1 SD – standard deviation

**Table 4.** The proportions of the gastrointestinal nematode third-stage larvae by species before and after treatment with pour-on eprinomectin and injectable ivermectin for the eight goat farms

Farm	D0 (%)						D14 (%)					
					Eprinomectin					Ivermectin		
	HC	TEL	TR	OE/CHAB	HC	TEL	TR	OE/CHAB	HC	TEL	TR	OE/CHAB
1	25	55	15	5	100	-	-	-	100	-	-	-
2	42	45	9	4	97	3	-	-	100	-	-	-
3	13	65	8	14	100	-	-	-	100	-	-	-
4	28	53	7	12	100	-	-	-	100	-	-	-
5	68	21	11	-	100	-	-	-	100	-	-	-
6	36	49	12	3	100	-	-	-	100	-	-	-
7	31	59	8	2	95	5	-	-	100	-	-	-
8	56	37	5	2	100	-	-	-	100	-	-	-

1 D0 – day of treatment; D14 – the 14^th^ day after treatment; HC – *Haemonchus contortus*; TEL – *Teladorsagia circumcincta*; TR – *Trichostrongylus* spp.; OE/CHAB – *Oesophagostomum/Chabertia* spp.

## Discussion

This study reports the first case of reduced efficacy of the pour-on formulation of EPN applied in goat herds in Slovakia. The results of the *in vivo* FECRT, however, could be divided into several categories with many possible interpretations. The EPG reduction differed little (1–3%) between pour-on EPN and the injectable form of IVM on half of the farms; these data could reflect the occurrence of GIN strains sensitive to ML anthelmintics, which was also confirmed by the FECRT values being slightly below 95% for both drugs. One herd even had a “normal” efficacy of EPN (96.60% with a 90% lower limit of the 95% confidence interval) according to the WAAVP guidelines (6). On one farm, however, both drugs had similar FECR%s of less than 90% (87.1% for EPN and 89.2% for IVM), which may be interpreted as an incipient case of cross-resistance to MLs, as previously described for moxidectin and IVM (25). The results differed on the remaining farms, where the FECRT values after application of pour-on EPN ranged from 51.0% to 69.4% and the percentage of EPG reduction was higher than after the administration of injectable IVM, ranging from 20% to 30%.

The number of similar studies reporting reduced efficacy of the pour-on EPN formulation has notably increased during the last decade. Resistance to EPN was described in a goat herd in Poland, where the percentage of EPG reduction after pour-on administration was between 0% and 20% (23). More than 40% difference in the FECRT results between pour-on EPN and an oral solution of moxidectin was reported on a goat farm in Austria, where the reductions in EPG after treatment with EPN and moxidectin were 44% and 86%, respectively (15). Several cases of reduced EPN efficacy have also been reported in France. Jouffroy *et al*. (17) observed this phenomenon for three routes of administration (pour-on, oral and subcutaneous) on five dairy sheep farms. Bordes *et al*. (2) recorded substantial differences in EPG reduction between moxidectin (100%), injectable EPN (21.5%) and pour-on EPN (-16.7%). We suppose that the slightly higher effectiveness of pour-on EPN (51.0%–96.6%) observed in our study is because the previous off-label use of EPN in Slovakia has been minimal because of its market unavailability and price barrier. Pour-on EPN nevertheless lacked efficacy on 87.5% (7/8) of the goat farms in the current study at the dose recommended for sheep and goats.

The dose of the pour-on formulation of EPN which is effective in small ruminants is still being actively discussed. Rostang *et al*. (27) suggested re-evaluating the recommended dose of EPN, mainly for lactating goats. Suboptimal dosing leads to the development of anthelmintic resistance (19, 28). The concentration of the drug that reaches the target GIN species in the host’s body can be affected by several factors, such as inter-individual variability, the pathophysiological status of the ruminant host and the route of drug administration (27). The area under the curve (AUC) that represents the extent of total exposure to the drug and its clearance rate from the host organism is considered an important indicator of ML efficacy. The AUC for pour-on EPN was shown to be highly variable among individuals, with the most substantial differences being between lactating and dry goats (10, 20). Similar pharmacokinetic variations for IVM in goats were described between the sexes (12) and different breeds (13). All these studies indicate that different breeds of animals and utility types and each sex may require individualised doses of the drugs for them to be effective. Another factor that can influence the effectiveness of the anthelmintic treatment is the species composition of the GINs on the farm.

In our study, *H. contortus* was the predominant GIN species after goat treatment (95%–100%), followed by *T. circumcincta* on two farms (3%–5%), similarly to other studies of the *in vivo* efficacy of EPN (2, 15, 17, 23, 24). Eprinomectin showed the lowest efficacy against *H. contortus* independently of external factors, even in an *in vitro* study (18), which could account for this agreement between results on several farms of small ruminants in Europe. *Haemonchus contortus* was most abundant on farms 2, 5 and 8 prior to treatment, where EPN had the lowest efficacy, indicating that *H. contortus* may be the species that resists EPN treatment the most and the critical indicator species for predicting EPN treatment efficacy. The *in vitro* LDT of our study could be performed only with IVM aglycone; therefore, the results of *in vivo* and *in vitro* efficacies of EPN cannot be directly compared. The data of the *in vitro* LDT, however, indicated an agreement between the *in vivo* and *in vitro* efficacies of IVM, because most of the GIN larvae that developed to the L3 stage at IVM concentrations equal to or higher than the MIC were on farms where the *in vivo* percentage in EPG reduction for IVM was below 90%. In contrast, the number of L3 larvae exposed to concentrations equal to or higher than the MIC was lower on farms where the effectiveness of IVM and EPN differed substantially. These data could indicate that the lower EPG reduction after treatment with EPN was not connected with the presence of resistant strains of GINs on farms, but with the insufficiency of the recommended dose of the drug to kill *H. contortus*.

## Conclusion

Eprinomectin remains the only anthelmintic registered for goats with no withdrawal periods for milk production. In future, the main goal for parasite control in goats will be to avoid a situation similar to that affecting the BZ anthelmintics, where resistant strains of GINs became globally widespread because of the most common faults in farm management and treatment schemes. An increasing number of studies indicate that the efficacy of EPN at the currently recommended dose for pour-on application is insufficient and may vary depending on internal and external factors. Further *in vivo* studies involving pharmacokinetic methods focusing on individual animal categories are required to clarify this issue.
